# Reducing Individual Variation for fMRI Studies in Children by Minimizing Template Related Errors

**DOI:** 10.1371/journal.pone.0134195

**Published:** 2015-07-24

**Authors:** Jian Weng, Shanshan Dong, Hongjian He, Feiyan Chen, Xiaogang Peng

**Affiliations:** 1 Bio-X Laboratory, Department of Physics, Zhejiang University, Hangzhou, Zhejiang, P.R. China; 2 Center for Brain Imaging Science and Technology, Department of Biomedical Engineering, Zhejiang University, Hangzhou, Zhejiang, P.R. China; 3 The First Hospital of Qiqihar, Qiqihar, Heilongjiang, P.R. China; Shenzhen institutes of advanced technology, CHINA

## Abstract

Spatial normalization is an essential process for group comparisons in functional MRI studies. In practice, there is a risk of normalization errors particularly in studies involving children, seniors or diseased populations and in regions with high individual variation. One way to minimize normalization errors is to create a study-specific template based on a large sample size. However, studies with a large sample size are not always feasible, particularly for children studies. The performance of templates with a small sample size has not been evaluated in fMRI studies in children. In the current study, this issue was encountered in a working memory task with 29 children in two groups. We compared the performance of different templates: a study-specific template created by the experimental population, a Chinese children template and the widely used adult MNI template. We observed distinct differences in the right orbitofrontal region among the three templates in between-group comparisons. The study-specific template and the Chinese children template were more sensitive for the detection of between-group differences in the orbitofrontal cortex than the MNI template. Proper templates could effectively reduce individual variation. Further analysis revealed a correlation between the BOLD contrast size and the norm index of the affine transformation matrix, i.e., the SFN, which characterizes the difference between a template and a native image and differs significantly across subjects. Thereby, we proposed and tested another method to reduce individual variation that included the SFN as a covariate in group-wise statistics. This correction exhibits outstanding performance in enhancing detection power in group-level tests. A training effect of abacus-based mental calculation was also demonstrated, with significantly elevated activation in the right orbitofrontal region that correlated with behavioral response time across subjects in the trained group.

## Introduction

Registering an individual brain to a template space is a common process in functional MRI (fMRI) studies and is known as the spatial normalization procedure. After spatial normalization, individual brain activation maps are projected into a standard coordinate space, enabling the comparison of voxel-wise functional activations across subjects. However, it has been reported that errors and bias always exist due to either distortion or signal loss during registration, which results from the differences between individual MRI images and the images representing the template [1–3]. It is difficult to achieve high accuracy in specific morphometric regions [1, 2, 4], such as the thalamus [2], parietal and frontal regions [1], regions around air-tissue interface. These artifacts cannot be eliminated completely and result in location misclassification and decrease sensitivity in group analysis [5]. Most available templates are based on adults. However, brain structure varies considerably in children, aging and some diseased populations [6]. Therefore, registration errors become more critical in studies of these specific populations. Not only does a child’s brain undergo massive changes in size and shape during maturation, but ongoing myelination of the white matter also changes, thus affecting the relative MRI contrast of gray and white matter [7, 8]. So it is a challenge to align children data perfectly to a common brain template generated from adults [9, 10]. Accordingly, the correction of errors and bias when registering children’s brains to a general template is of great importance [9, 11].

One way to explicitly account for this challenge is to normalize group data (such as children groups) to a population-specific template (such as a children template), or even to a study-specific template. The usage of a population-specific or a study-specific template has been recommended to minimize registration errors and improve sensitivity in statistical analyses [1, 5]. For children data, a population-specific or study-specific template might better represent brain shape, size and tissue concentration and reduce subjects’ brain variations compared to adult templates. Consequently, children’s templates have been created based on a large sample over 50 subjects per group. Infant brain templates from neonates to 1- and 2-years-old have been developed based on 95 normal infants [12]. A Japanese children template was developed based on 180 children between the ages of 5 and 9 years [13]. Recently, Luo et al constructed a brain template for the Chinese pediatric population based on 53 children [6]. In these studies, a sufficiently large sample size is required to capture sufficient group-level variation to create a representative template. However, a large sample size is not always obtained easily in reality, especially more difficult for children studies. Because there lacks evaluation of a template based on a small sample size, the first aim of the present study was to quantify the impact of a study-specific template with a small sample size on normalization.

In addition to creating specific templates, researchers also tried to minimize possible influence of individual variation during normalization by proper correction [1, 14]. For instance, a 12-parameter affine transformation that contains translation, rotation, scaling and shearing is usually applied as one of normalization steps. This process aims to translate an image from the native space to a standard space defined by a given template. This affine transformation matrix carries critical information about individual variability related with brain anatomy. Previous volumetric studies have determined that structural variation correction (such as head size) derived from the affine transformation matrix might increase the robustness of normalization results [1, 14]. Therefore, it is reasonable that a proper correction based on the affine transformation matrix might reduce individual variation related to registration bias and improve the registration result. We assume that this registration improvement might also improve group-level results in fMRI studies. Thus, the second aim of the current study was to reduce registration errors by brain structural variation correction based on the affine transformation matrix.

The distance or similarity between a native image and a template can be represented by the Euclidean difference [15]. The Euclidean difference would arise with increasing differences of images. A previous study concluded empirically that the Frobenius norm (FN) yielded the best result to measure the Euclidean difference [16]. The FN of the affine transformation matrix is defined as the sum of the absolute squares of its elements in linear algebra [17]. These measurements and similar variants have been used extensively to capture the Euclidean difference between a template and a native brain image, and to evaluate the differences between images [18]. We adopted this idea and used its square root (SFN) in the current study of children. Because the component of translation in the matrix is unrelated to the change in anatomical geometry, it was not included in the calculation. In addition, the Frobenius norm has the invariant property of unitary rotation transformation. Consequently, the defined SFN only took into account the transformation of scaling and shearing operations. This is consistent with our interests in brain structure instead of spatial position. We hypothesized that the SFN values might capture between-image differences in brain structure. Thus, a correction based on the SFN might reduce individual variation and group normalization bias. To the best of our knowledge, this is the first study to evaluate the effect of brain anatomic variation correction on normalization in an fMRI study.

As part of the prefrontal cortex, the orbitofrontal cortex (OFC) is particularly sensitive to imaging signal dropout and distortion. The variable morphology of the OFC also complicates accurate alignment in image registration processes [4]. Functionally, the OFC takes part in a multitude of cognitive processes, including decision-making [19, 20] and working memory [21, 22]. The OFC also receives visual input from the inferior temporal visual cortex to represent objects (such as facial expression) and identity [23, 24]. This region may be an essential hub for processing image information and converting it into other types of information [23, 24]. In sum, it seems practical to minimize registration errors introduced by normalization processes and improve activity detection in the OFC.

The current children’s fMRI study had two main goals. The first was to explore the performance of a group template based on a small sample size. The population of this study was 29 Chinese children divided into two groups: an abacus-trained group and a control group. Evidence has shown that a population-specific template improves the characterization of the brain structure of children participants [9]. We hypothesized that a study-specific group-averaged template from our extremely limited number of subjects might also capture these variations, and improve the normalization process by decreasing registration errors. We compared the performance of three different templates: the Western adult MNI template (MNI), a Chinese children population-specific template (CCT) constructed using age-matched Chinese children [6], and a study-specific template (SST) created from the average over all subjects in this study as performed previously by Huang et al [5]. The second goal was to verify the assumption that the SFN is a factor that can be used to characterize individual registration-related variation. In this study, the SFN derived from affine transformation in normalization procedure was employed as a covariate in group analysis for further analysis. We expected that proper template choice in normalization procedure and the inclusion of SFN as a covariance in group analysis would reduce individual variation and enhance detection power in the group-level tests.

## Methods

### Ethics statement

The experimental protocol and informed consent for this study were approved by the research ethics review board of Zhejiang University in China. All children and their parents were provided informed written consent and signed it before the experiment. We acquired signed written consent to publish their MRI images.

### Participants

Twenty-nine healthy children (8.01±0.59 years, 15 males) participated in this study on the training effect of abacus-based mental calculation (AMC). These young volunteers from a single district of Qiqihar city in China constituted two groups: sixteen in a trained group (7.92±0.67 years, 9 males; 7.88±0.85 years, 7 females) with one-year of AMC training, and thirteen children in a control group (8.17±0.37 years, 6 males; 7.88±0.44 years, 7 females) without any abacus knowledge. The AMC group was able to perform calculations with no physical abacus but by mentally visualizing the abacus. It is believed this training system triggers a new strategy to process numbers [25]. The two groups were matched with respect to other educational background.

### Experimental design

**Fig 1:**
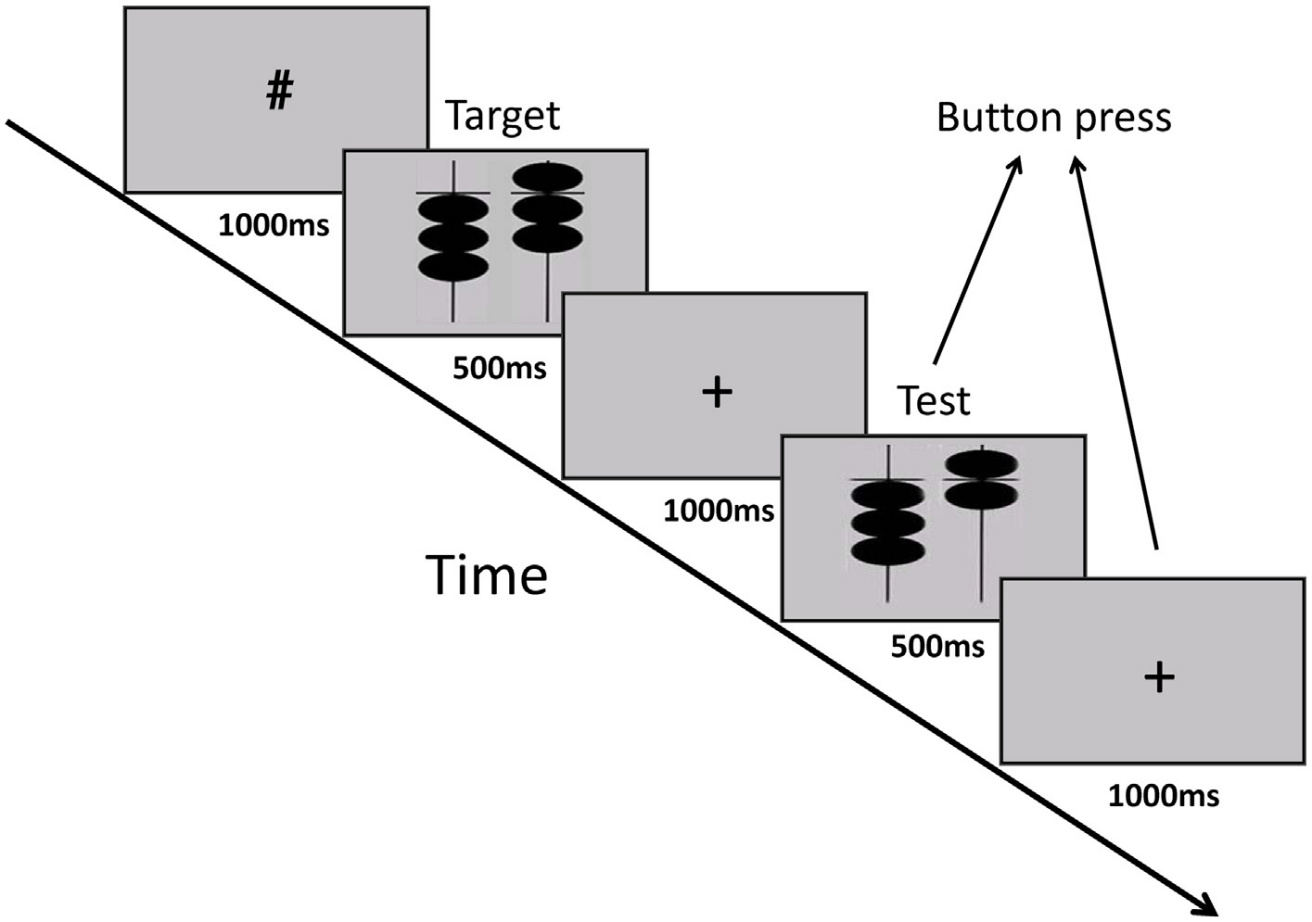
Illustration of the bead-picture match task. Schematic illustration of 1 trial of the experiment design, the bead-picture match task.

The volunteers were asked to perform a block-designed working memory task to compare two pictures (Fig 1). The paradigm consisted of nine blocks, and each block had a 24-s task condition alternating with a 16-s rest condition. Every task block included six trials of 4 s each. Each trial started with a ‘#’ for 1000 ms, followed by an abacus-bead picture for 500 ms, a fixation cross ‘+’ for the next 1000 ms, a second abacus-bead picture for 500 ms, and another fixation cross ‘+’ for the final 1000 ms. The subjects were asked to compare the two bead pictures in the scanner, decide whether they were identical, and respond by pressing a button as soon as possible after the second abacus-bead picture was presented. This task is based on the classic n-back task, which is a widely used paradigm in cognitive neuroscience to assess working memory [26]. Detailed information about this task was reported in our previous fMRI study [27]. The behavioral response time was recorded and used in the correlation analysis with the BOLD signal. The resting-state block had only a fixation cross ‘+’ on the screen for the entire 16 s period.

### MRI acquisition

MR data were acquired on a 1.5-T MRI clinical scanner (Achieva, Philips) with an 8-channel head coil in the first hospital of Qiqihar city. The functional images were collected using a single-shot gradient-echo echo-planar imaging (EPI) sequence with the following parameters: TR/TE = 2000/50 ms, flip angle = 90°, FOV = 230 mm × 230 mm, matrix = 64 × 64, slice thickness/gap = 5 mm/0.8 mm, and 22 slices in an interleaved ascending order to cover the whole brain. These images were set obliquely and parallel to the anterior and posterior commissure line. The entire functional scan included 180 measurements and lasted for 360 s. Another high-resolution anatomical scan was also acquired for each subject using a 3D fast field echo (FFE) sequence with the following parameters: TR/TE = 25/4.6 ms, flip angle = 15°, FOV = 256 mm × 256 mm, acquisition matrix = 256×256, reconstruction voxel size = 1 × 1× 1 mm^3^, 150 slices in the sagittal plane.

### Data pre-processing

Imaging processing was performed using SPM8 (http://www.fil.ion.ucl.ac.uk/spm) on Matlab (version 2010b; The MathWorks, Natick, MA, USA). The first three scans were discarded to remove the impact of magnetization stabilization. Data were subsequently corrected for slice-timing differences. Functional image volumes were then registered and aligned to the first time point using a rigid-body transformation to correct head motion. Although it was difficult for our young volunteers to remain motionless during the task, none were found to have head motion greater than 2.0 mm in displacement or 2° in rotation in any direction. After motion-correction, it was necessary to perform spatial normalization and transform the native brain image into a standard template for subsequent voxel-wise group comparisons. During normalization, an individual’s native anatomical image was registered into a template space, and the transformation was applied to EPI images. This transformation involved a 12-parameter linear affine transformation followed by non-linear deformation [28, 29]. Three templates were adopted separately in this procedure, and it was our primary purpose to evaluate the performance of the template based on a small sample size. The templates included the standard MNI template (MNI) from the SPM package, a Chinese children template (CCT) proposed by Luo et al that claims to better represent the Chinese children population [6], and a study-specific template (SST) obtained from the average of all subjects in this study, as performed previously by Huang et al [5]. All three templates followed the MNI coordinate to enable a comparison of the activation results among templates. The normalized images were further smoothed with a Gaussian kernel of 6 mm full width at half maximum to enhance the signal-to-noise ratio.

### SFN

The SFN value of the affine transformation matrix was used to measure the Euclidean difference between the template and a native brain. The initial transformation matrix obtained from normalization in SPM was a 4 × 4 square matrix. As mentioned above, the last column and row reflecting the translation part were discarded, and the SFN was derived from the remaining 3 × 3 matrix. By definition, the SFN was equal to the sum of the absolute squares of the matrix elements. We used the norm function with the type of ‘fro’ in Matlab, and took its square value as the SFN for implementation.

We analyzed the dependent measures of the SFN by employing repeated ANOVAs, including Templates (three templates: MNI, CCT, and SST) as a within-subject factor and Groups (trained and control) as a between-subject factor. Greenhouse-Geisser adjustments were used if sphericity could not be assumed. Follow-up post-hoc multiple comparisons were computed using Bonferroni correction.

### Statistical process

Task activation was estimated using a two-level statistical model in SPM. In the first level, individual activation maps were computed with a general linear model [30]. A high-pass filter with a cut-off frequency of 1/128 Hz was applied to remove slow drift. Head-motion parameters were treated as confounds and included as covariates in the linear regression. For each subject, the effect of functional task was then estimated, and the contrast of the task condition was obtained. Group-level comparisons were then conducted with individual contrasts to determine within-group activation by one-sample t-test and between-group differences by two-sample t-test. Furthermore, these group comparisons were also re-analyzed with the SFN included as a covariate as a source of individual variability. The SFNs were separated into two groups and centered on the mean value of each group.

AlphaSim correction was applied in the final activation maps to control the false-positive rate with thresholds of adjusted *p* < 0.05 (corresponding to voxel-wise threshold of *p* < 0.01, FWHM = 6 mm) and a cluster size of 34 voxels. This correction was computed using the REST program (http://restfmri.net/forum/rest) [31].

### Region of interest (ROI) analysis

The right OFC (rOFC) was of particular interest in this study. The ROIs of the OFC were defined in two ways. For the correlation analysis between the SFN and regional BOLD contrast size, both the left and right ROIs were defined from the AAL template. The averaged BOLD contrast size for each subject was calculated from the selected ROI. In a further investigation of the relationship between BOLD activity and behavioral response time, we selected the significant cluster by between-group differences inside of the rOFC.

The significant relationships reported in this correlation analysis remained if the outliers (Cook’s distance > 1) were excluded from the correlation analyses, suggesting that these correlations were not a statistical artifact.

## Results

**Fig 2:**
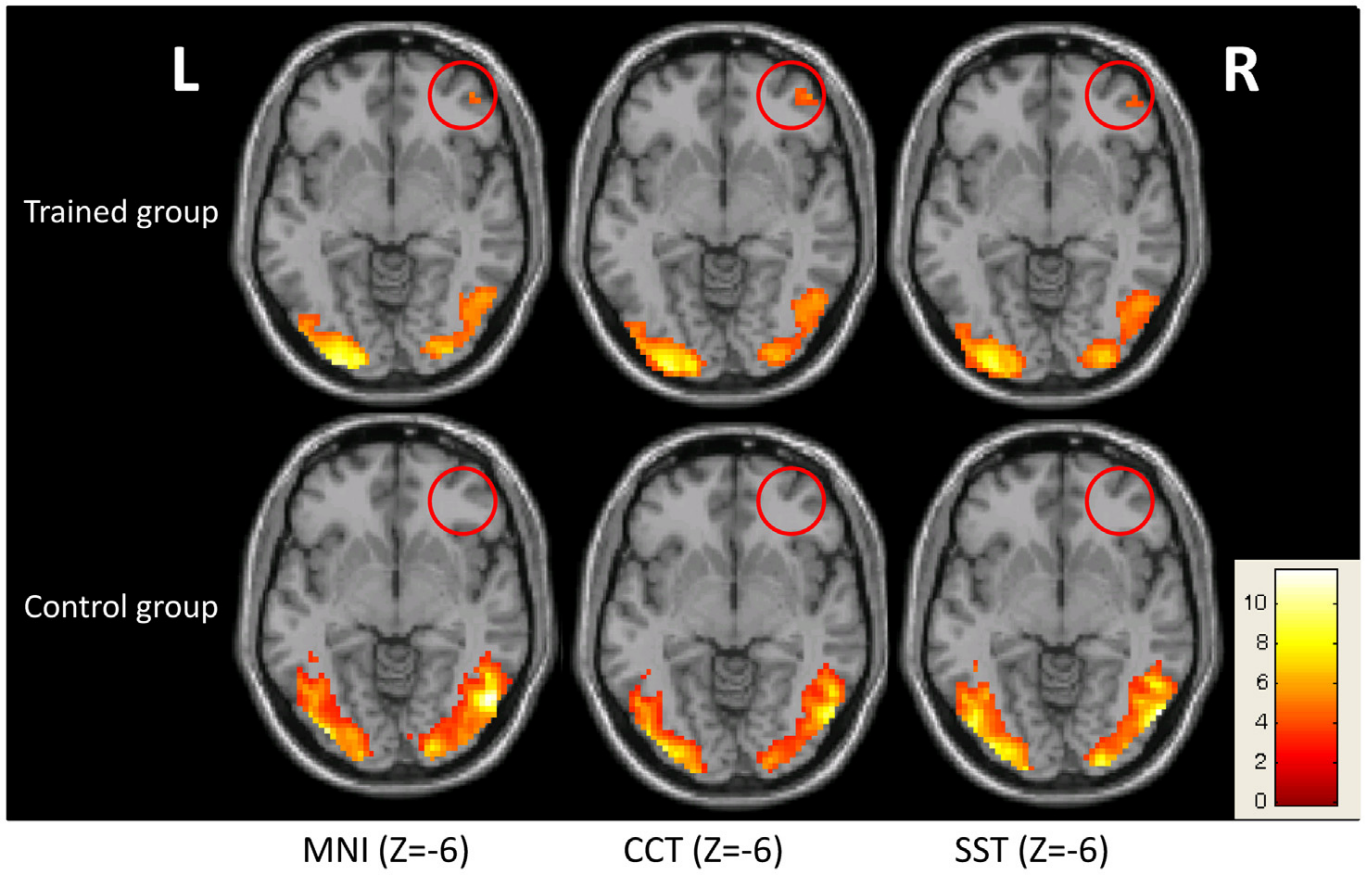
Active regions during the bead-picture match task. Regions activated during the bead-picture match task in each group (*p* < 0.05, AlphaSim corrected). Activation in the rOFC (marked in red circle) was only observed in the trained group, and not in the control group.

A series of common regions were activated in both two groups, including the bilateral inferior occipital gyrus and left fusiform gyrus. These regions are necessary for a typical picture identification task. More interestingly, activation in the rOFC was only detected in our trained group. This observation was consistent across all three templates used for normalization, with minor observable differences in cluster size (Fig 2, *p* < 0.05, AlphaSim corrected).

**Fig 3:**
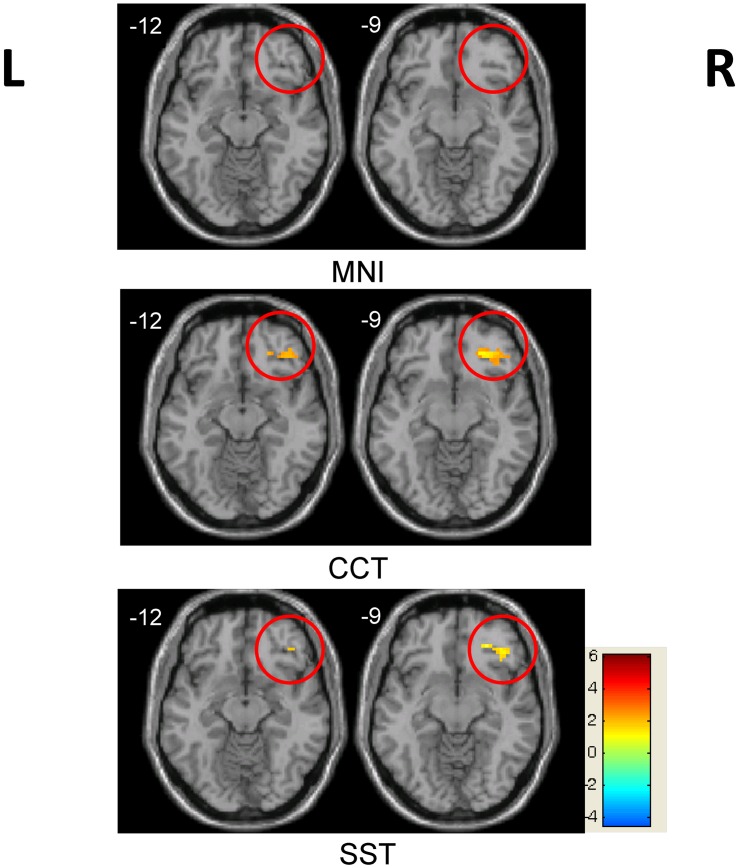
Between-group differences in the rOFC region across three templates. Significant between-group differences in the rOFC region (marked by red circle) were observed using both the CCT and SST templates, but not the MNI template (*p* < 0.05, AlphaSim corrected). Red indicates trained group > control group; blue indicates trained group < control group.

This difference was further supported by the between-group comparison (Table 1A). Two sample t-tests revealed the greater activation of bilateral inferior occipital and left fusiform regions in the control group, whereas activation in regions such as the rOFC, right inferior parietal lobule and left cingulated gyrus was significantly greater in the trained group. Importantly, the increase in the rOFC was only detectable using CCT or SST, but failed to reach significance using the MNI template (Fig 3). This result indicates that CCT and SST performed better in our children study than the adult MNI template.

**Table 1:** Regions of significant between-group differences, coordinates (x, y, z) and T-values of local maxima detected across three templates for the bead-picture match task without and with SFN correction (*p* < 0.05, AlphaSim corrected).

	MNI				CCT				SST			
Active regions	x	y	z	T-value	x	y	z	T-value	x	y	z	T-value
**A. No covariates**												
**Trained > Control**												
L cingulate gyrus	-15	-18	33	4.58	-18	-24	48	3.72	-15	-15	36	3.91
R inferior parietal lobule	39	-27	27	6.68	42	-30	30	5.40	42	-30	30	4.50
R frontal inferior orbital					30	39	-6	3.90	27	39	-9	3.38
**Control > Trained**												
L inferior occipital gyrus	-36	-84	-6	4.21	-36	-87	-6	4.16	-33	-81	-9	3.66
L fusiform	-48	-57	-12	4.27	-39	-63	-12	3.14	-33	-60	-12	3.00
R inferior occipital gyrus	45	-48	-24	4.40	45	-51	-24	4.75	42	-48	-27	4.70
**B. SFN regarded as a covariate**												
**Trained > Control**												
L cingulate gyrus	-15	-18	33	4.54	-18	-24	48	3.75	-15	-15	39	3.90
L frontal inferior orbital					-30	42	-3	4.15				
R inferior parietal lobule	39	-27	27	6.78	42	-30	27	5.72	42	-30	30	4.55
R frontal inferior orbital	27	39	-6	3.44	27	39	-6	4.44	27	39	-9	3.80
**Control > Trained**												
L inferior occipital gyrus	-48	-75	-12	4.19	-30	-84	-9	4.18	-30	-81	-9	3.65
L fusiform	-48	-57	-12	4.29	-42	-63	-12	3.17	-36	-51	-15	3.38
R inferior occipital gyrus	30	-45	-12	4.48	45	-51	-24	4.60	42	-48	-24	5.09

We hypothesized that the SFN might be useful to characterize the differences in brain anatomy between images. For each subject, the SFN values were calculated in both groups for the three templates respectively (Table 2). A 3 (Templates: MNI, CCT or SST) × 2 (Groups: trained or control) repeated ANOVA on the mean SFN yielded a main effect of Templates, F (1.111) = 176.40, *p* < 0.001, partial η^2^ = 0.87. Post-hoc analysis revealed that the SFNs of the MNI template were significantly larger than the CCT and SST templates (*p*<0.001, Bonferroni correction). These data suggest that the use of MNI template in normalization always gives larger SFNs. Meanwhile, no significant interaction effect between Templates and Groups was detected (F (1.111) = 0.13, *p* < 0.746, partial η^2^ = 0.01), indicating that there were no significant differences of the SFNs between the trained and control group in all three templates. This is consistent with our expectation because all subjects were well-matched.

**Table 2:** Mean (±Standard Deviation) of the SFN in the two groups across the three templates.

	MNI	CCT	SST
Trained Group	4.24 (0.22)	4.12 (0.20)	3.80 (0.24)
Control Group	4.15 (0.15)	4.06 (0.16)	3.73 (0.15)

**Fig 4:**
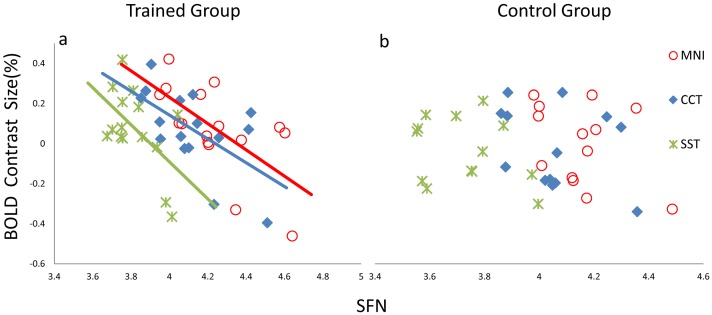
Correlations between BOLD contrast size and the SFN in the rOFC. Scatter plot showing the distribution of BOLD contrast size in the rOFC as a function of the SFN. (a) The SFN was significantly correlated with BOLD contrast size (*p* < 0.05) among the three templates in the trained group (MNI: *r* = -0.65, *p* = 0.006; CCT: *r* = -0.59, *p* = 0.015; SST*: *r* = -0.54, *p* = 0.036). (b) Scatter plot revealing that the SFN had no effect on BOLD contrast size among the three templates in the control group (MNI: *r* = -0.30, *p* = 0.320; CCT: *r* = -0.40, *p* = 0.177; SST: *r* = -0.29, *p* = 0.338). (* one subject had much smaller SFN (3.48) than others in the trained group (mean SFN 3.82), but there was no difference when all subjects were considering. This subject’s data were not included in this correlation analysis.)

**Fig 5:**
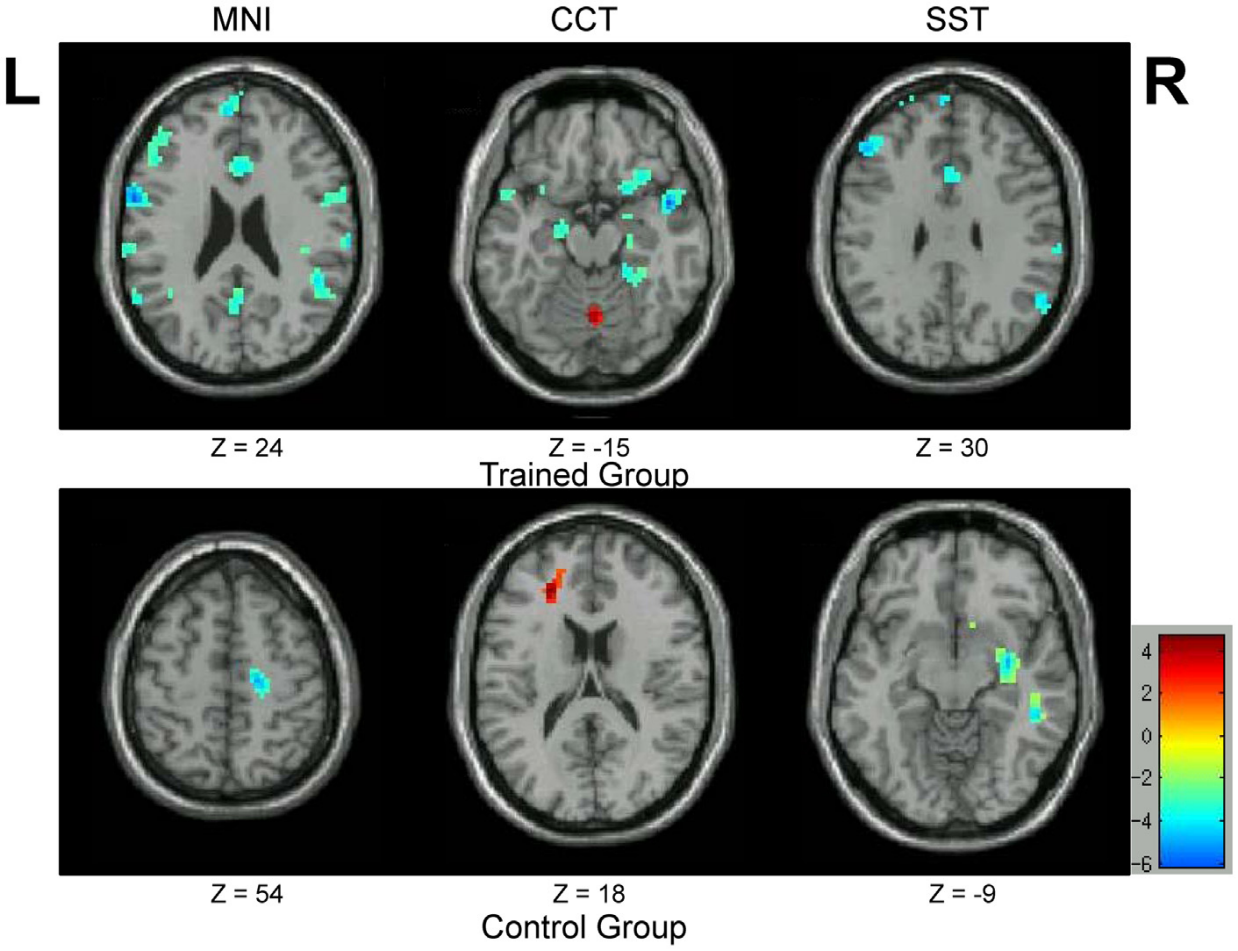
Whole-brain correlation maps of the SFN and BOLD contrast size. Voxel-based whole-brain correlation maps of the SFN and BOLD contrast size (*p* < 0.05, AlphaSim corrected) among the three templates.

**Fig 6:**
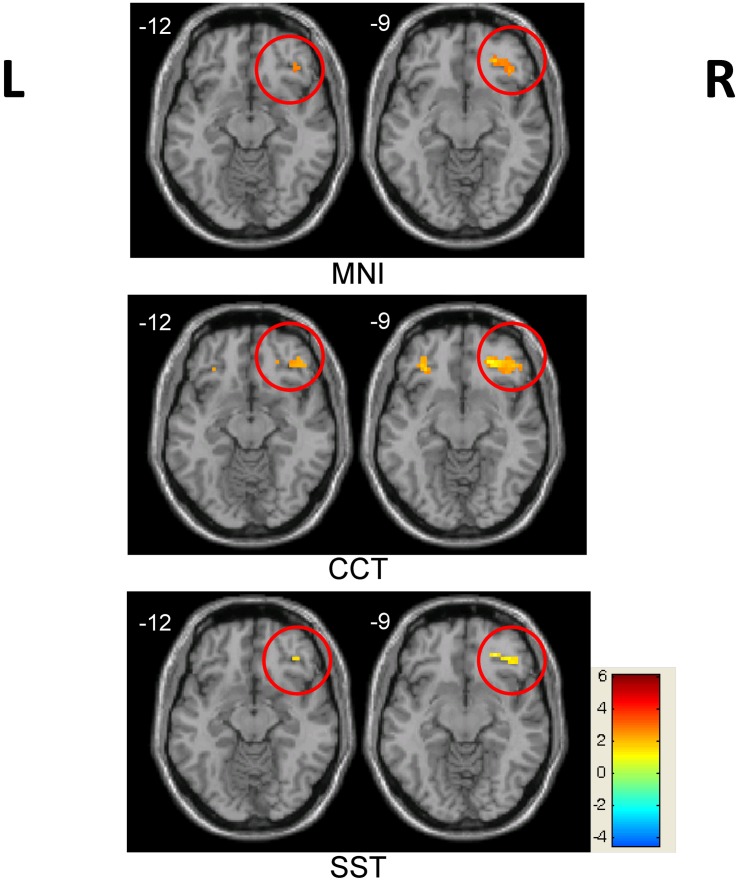
Between-group differences after SFN correction. Group differences in the rOFC region (marked) were consistently detected for all three templates when the SFN was included as a covariate (*p* < 0.05, AlphaSim corrected). Red indicates trained group > control group; blue indicates trained group < control group.

Further investigation demonstrated that BOLD contrast size was highly negatively correlated with SFNs in the rOFC across subjects in the trained group, which was consistent in all three template conditions (MNI: *r* = -0.65, *p* = 0.006; CCT: *r* = -0.59, *p* = 0.015; SST*: *r* = -0.54, *p* = 0.036). However, this correlation was only observed in the trained group and not in the control group (MNI: *r* = -0.30, *p* = 0.320; CCT: *r* = -0.40, *p* = 0.177; SST: *r* = -0.29, *p* = 0.338) (Fig 4). No significant correlation was observed in the left OFC in either group, which suggests that the SFN may have a greater impact on the activated region. As a further verification, voxel-wise whole-brain analysis revealed that more regions showed significant correlation between BOLD contrast size and the SFNs (Fig 5, *p* < 0.05, AlphaSim corrected), most of which were activated or deactivated during the task. When the SFN was considered a covariate to control for individual variation and employed in the group comparisons, activation in the rOFC was consistently observed in all three templates (Fig 6 and Table 1B). This enhancement of activation, particularly with the MNI template, confirms our assumption that the SFN correction might reduce normalization bias in group analysis and improve activation sensitivity.

**Fig 7:**
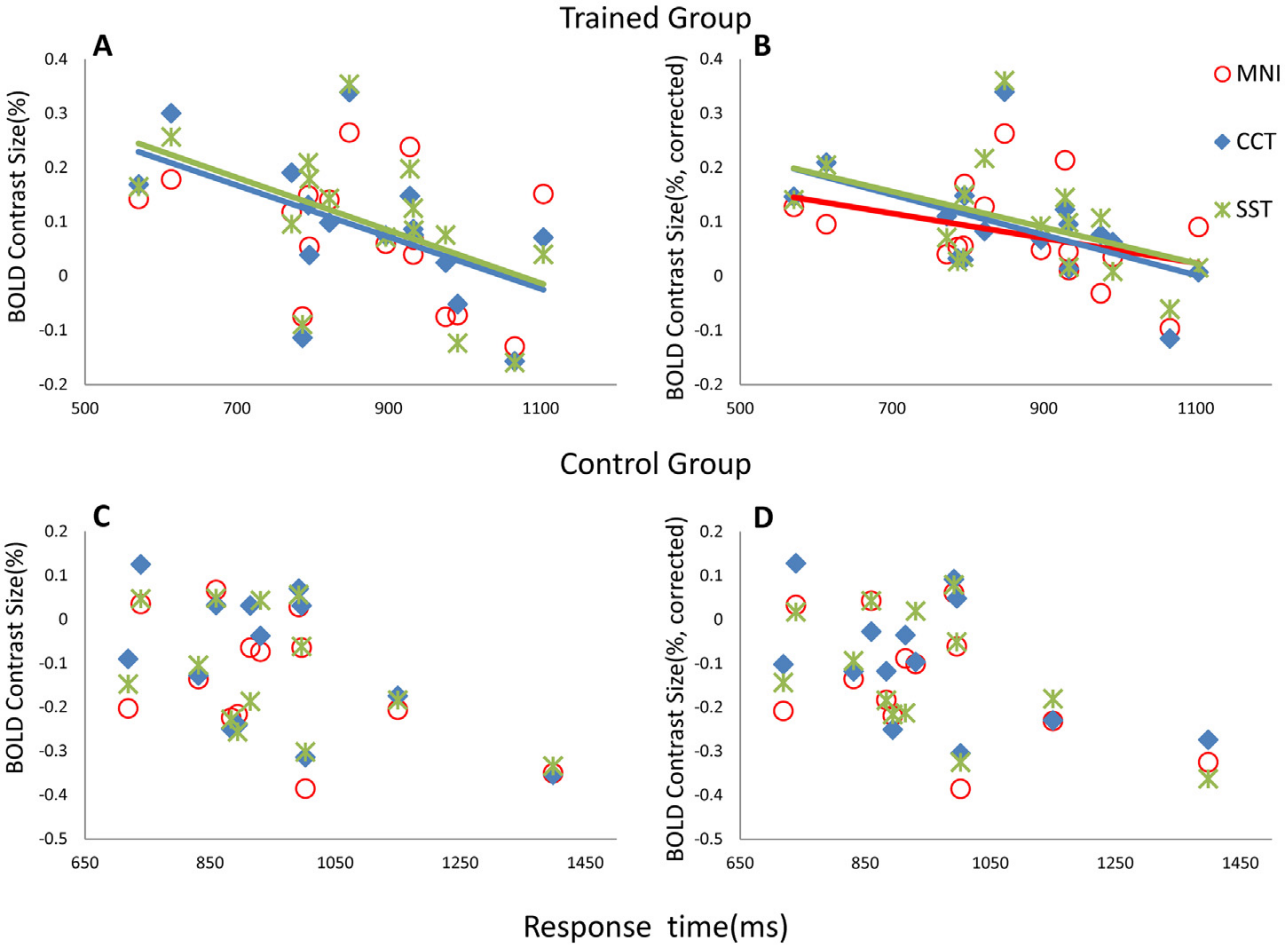
Relationship between response time and BOLD contrast size in the rOFC. The relationship between the subject’s response time in the trained group and BOLD contrast size in the rOFC were correlated (*p* < 0.05) for the CCT (*r* = -0.54, *p* = 0.019) and SST (*r* = -0.51, *p* = 0.023) templates, but not for the MNI template (*r* = -0.39, *p* = 0.192) **(a)**. After SFN correction, significant correlations were observed across all three templates in the trained group (MNI: *r* = -0.59, *p* = 0.017; CCT: *r* = -0.59, *p* = 0.016; SST: *r* = -0.57, *p* = 0.02) **(b)**. However, no negative correlation was observed in the control group without (MNI: *r* = -0.48, *p* = 0.119; CCT: *r* = -0.52, *p* = 0.087; SST: *r* = -0.47, *p* = 0.151) or with (MNI: *r* = -0.46, *p* = 0.115; CCT: *r* = -0.49, *p* = 0.091; SST: *r* = -0.51, *p* = 0.077) SFN correction, regardless of the brain template used **(c, d)**.

In the bead-picture match task, the response time (RT) was analyzed in each group. The mean (±Standard Deviation) response time was 864 (± 145) ms in the trained group and 947 (± 177) ms in the control group. A Student two-sample t-test on RT indicated that there was no significant difference between the two groups (t(27) = -1.385, *p* = 0.177). The relationship between BOLD contrast size and the children’s behavior response time during the task was also investigated. The two terms were correlated in the trained group when CCT (*r* = -0.54, *p* = 0.019) or SST (*r* = -0.51, *p* = 0.023) were applied during normalization but not when the MNI template was used (*r* = -0.39, *p* = 0.192) (Fig 7a). The negative relationship was more consistent, with the same threshold after SFN correction (MNI: *r* = -0.59, *p* = 0.017; CCT: *r* = -0.59, *p* = 0.016; SST: *r* = -0.57, *p* = 0.02) (Fig 7b). There was no correlation detected in the control group, regardless of the template used, without SFN correction (MNI: *r* = -0.48, *p* = 0.119; CCT: *r* = -0.52, *p* = 0.087; SST: *r* = -0.47, *p* = 0.151), or with SFN correction (MNI: *r* = -0.46, *p* = 0.115; CCT: *r* = -0.49, *p* = 0.091; SST: *r* = -0.51, *p* = 0.077) (Fig 7c and 7d). The elevated activation in the rOFC and its correlation with response time likely reflect the biological difference between trained and control children in their processing of the bead-picture match task.

## Discussion

This study suggested two approaches to reduce individual variation and improve normalization results: proper template choice and SFN correction. We evaluated the performance and accuracy of the SST template, based on a small sample size, in the normalization process. Our results indicate that group activation differences are more robustly and sensitively detected with the CCT and SST templates than the standard adult MNI template. We suggest that it is feasible and advantageous to create a study-specific template, even with a small sample size. We also demonstrated that the SFN can capture the errors induced by linear transformation and can be used as a covariate for inter-subject correction. After correction, the results of activation and correlation become more consistent across the three templates.

### Index of SFN

We determined that the SFN differed significantly depending on the template used during normalization. The SFN was generated from an affine transformation matrix and defined as the similarity between each individual image and the template. We further investigated whether the SFN affected the BOLD contrast size. Whole-brain analysis revealed significant correlations between the SFN and BOLD contrast size across all subjects for the three templates respectively (Fig 5), and the most correlated voxels were located near the tissue boundaries and in regions in the frontal lobe and cerebellum. The area of the OFC was also included (Fig 4a). The biological meaning of this correlation is unclear, but it is most like errors and bias introduced by image registration. Interestingly, in contrast to the MNI template, the correlations between the SFN and BOLD contrast size in the rOFC were weaker when CCT and SST were used in normalization (Fig 4). On the other hand, we also observed that the SFNs were significantly smaller in the CCT and SST than those in the MNI template. The degree of similarity between the individual and template is inversely proportional to the SFN values [32]. Our results show that employing CCT and SST in the normalization process could increase the similarity between individual images and the template and improve the characterization of children’s brain structure compared to the MNI template. Consequently, registration bias might increase when the MNI adult template is used for fMRI analysis in children, compared to CCT and SST, in line with a previous volumetric study [1]. Therefore, it might be feasible to use a study-specific template, even if the study population has limited sample, or to use a population-matched template to control and minimize the influence of registration errors associated with template usage.

### Template choice and SFN correction

We hypothesized that a study-specific brain template of small sample size could increase the detection power of between-group activation differences. In our bead-picture match task, co-localizing activations were detected, including the bilateral inferior occipital gyrus, left fusiform, right inferior parietal lobule and left cingulate gyrus, in all three templates. However, the child-based templates, SST and CCT, detected between-group activation differences in regions of the rOFC; such differences were not observed when the adult-based MNI template was utilized. The observed activation differences in the rOFC are due solely to the use of a different template in the normalization procedure. The MNI template is an average brain template constructed from 305 young Western adult brains. However, our volunteers were all children from a single region of China, whose brains differ considerably in shape and size from Western adult brains. In line with previous studies [5, 33], our study demonstrated that a population-matched template (CCT) reduced registration errors and improved the results. Importantly, the CCT and SST produced very similar results. It is remarkable that the SST from a small sample was able to capture brain characteristics and exhibit superior performance in our children data analysis.

Due to registration errors and bias would be introduced by individual variation, it is necessary to characterize anatomic differences and minimize individual variation in study populations. The SFN was introduced and evaluated as an individual index in this study. Our study shows that using the SFN as a covariate is practical in group statistics. As an example, group activity was consistently in the rOFC regardless of the template used after SFN was introduced as a covariate in the design matrix. This result is consistent with previous morphometric analyses using specific statistical correction procedures that suggested that employing brain anatomic information as a covariate in group analyses may be valuable in specific populations [34, 35]. Another study also reported that gray-matter volume changes in aging could be detected after whole-brain anatomic difference correction [36], a finding replicated in our children functional data. Thus, our findings suggest that inclusion of the SFN as a covariate might be effective for reducing the effect of individual variation on fMRI results in group analyses and enhance the detection power in group statistics. Interestingly, significant between-group differences were identified in the left frontal inferior orbital region, only when the SFN was included as a covariate in the CCT template. This difference could only be detected with an uncorrected P-value of 0.05 when the two other templates were applied. This could probably be the particular merit provided by CCT, which is based on a larger cohort of subjects than SST.

Another major evidence of the current study is about the robustness of the correlation results between regional BOLD activity and behavioral response time across subjects. We observed an enhancement of the correlation strength in the trained group after adopting a proper template for normalization or after employing the SFN correction (Fig 7a and 7b). Note that this correlation was not sufficiently strong to reach a significance level of *p <* 0.05 when a traditional MNI template was applied. This suggests the importance of a proper brain template choice in children data analysis. Large differences between individual brains and the template increase the risks of misalignment and inaccuracy in the registration process. Our results also demonstrated that the SFN correction might sufficiently enrich the power of statistical analyses. The plausible result of an improved negative correlation in the rOFC under all three templates confirms usefulness of this simple correction. Taken together, these observations demonstrate the importance of template selection and post-hoc correction with the SFN.

### Right OFC

The major findings in this study are associated with the rOFC. Optimizing the normalization process and group statistics increased our confidence in the group activation differences between the trained and control groups. Specifically, the one sample t-test in Fig 2 demonstrates that the rOFC was only activated in the trained group, and not the control. Further investigation revealed that individual BOLD amplitudes in the trained group were correlated with the average response times during the task. No significant correlation was observed in the control group, who had no experience with an abacus. The BOLD signal changes in the rOFC of control group were near zero, with a tendency to be negative. It indicates that this region was not involved when the controls were performing the task during functional scans. Hence, it is no surprise that the BOLD contrast size and behavior scores were not correlated in this region for the control group, even after our proposed correction (Fig 7c and 7d). By contrast, the rOFC was one of the activated nodes in the trained group, as shown in Fig 2, and was also a major point of difference between these two groups (Figs 3 and 6 and Table 1).

Our results confirm that the two groups, with or without abacus training, might use a different strategy when performing our bead-picture match task, and this conclusion can be emerged from the results of between-group activation differences in the rOFC. In the trained group, subjects have been trained to master the abacus for arithmetic when beads or digits are displayed. In the bead-picture match task, trained subjects might process bead image information by automatically converting it into digit information before completing the task. This automatic strategy has been demonstrated closely related to a visuospatial process [24]. Previous studies have observed functional differences between trained and control groups in the right hemisphere, and suggested the lateralization of the non-verbal AMC strategy [26]. These prior results are consistent with our finding of significant differences in the rOFC (Fig 2). In addition, there was a significant negative correlation between BOLD contrast size in the rOFC and behavior performance across subjects only in the trained group (Fig 7). The negative correlation indicates that subjects with better performance utilized more cognitive resources. Therefore, our findings of between-group activation differences and correlation results in the rOFC are not artifacts of this study. Our data emphasize the important role of the rOFC in our bead-picture match task in the trained AMC group.

## Conclusion

Normalization to a template such as MNI in SPM is a common process for group studies. Registration error is a crucial source of individual variation, and should be minimized. Firstly, our study verified the importance of template selection on group-level results. This result is consistent with previous findings that study-specific templates based on a sufficient sample size perform better in normalization procedures [1, 5]. Importantly, our results show that study-specific templates with a relatively small sample size might provide a significant improvement in children MRI data analysis. Secondly, to the best of our knowledge, this is the first study to propose the use of the SFN to characterize the differences between native brains and templates in an fMRI study. The usage of SFN as a covariate in group analysis might successfully capture these differences and reduce the registration errors in activated regions such as the OFC. Both approaches could effectively reduce individual variation and enhance detection power in group-level tests in fMRI studies with small sample sizes. Finally, the robust activation in the OFC and its negative correlation with behavior performance in the trained group reasonably indicates that the OFC is critical for mental abacus training and merits further investigation.
